# Carbon Monoxide Potentiates High Temperature-Induced Nicotine Biosynthesis in Tobacco

**DOI:** 10.3390/ijms19010188

**Published:** 2018-01-08

**Authors:** Tielong Cheng, Liwei Hu, Pengkai Wang, Xiuyan Yang, Ye Peng, Ye Lu, Jinhui Chen, Jisen Shi

**Affiliations:** 1 Co-Innovation Center for the Sustainable Forestry in Southern China, Nanjing 210037, China; chengtl@njfu.edu.cn (T.C.); pengye@njfu.edu.cn (Y.P.); luye@njfu.edu.cn (Y.L.); chenjh@njfu.edu.cn (J.C.); 2 College of Biology and the Environment, Nanjing Forestry University, Nanjing 210037, China; 3 Laboratory of Tobacco Agriculture, Zhengzhou Tobacco Research Institute of CNTC, Zhengzhou 450001, China; hulw@ztri.com.cn; 4 Key Laboratory of Forest Genetics and Biotechnology, Ministry of Education, Nanjing Forestry University, Nanjing 210037, China; pm@ism.cams.cn; 5 Research Center of Saline and Alkali Land of State Forestry Administration, China Academy of Forestry, Beijing 100091, China; yangxiuyan@caf.ac.cn

**Keywords:** carbon monoxide, high temperature, nicotine, jasmonic acid, tobacco

## Abstract

Carbon monoxide (CO) acts as an important signal in many physiological responses in plants, but its role in plant secondary metabolism is still unknown. Nicotine is the main alkaloid generated in tobacco and the plant hormone jasmonic acid (JA) has previously been reported to efficiently induce its biosynthesis. Whether and how CO interacts with JA to regulate nicotine biosynthesis in tobacco remains elusive. In this study, we demonstrate that high temperature (HT) induces quick accumulation of nicotine in tobacco roots, combined with an increase in CO and JA concentration. Suppressing CO generation reduced both JA and nicotine biosynthesis, whereas exogenous application of CO increased JA and nicotine content. CO causes an increased expression of *NtPMT1* (a key nicotine biosynthesis enzyme), via promoting NtMYC2a binding to the G-box region of its promoter, leading to heightened nicotine levels under HT conditions. These data suggest a novel function for CO in stimulating nicotine biosynthesis in tobacco under HT stress, through a JA signal.

## 1. Introduction

The low molecular weight diatomic gas carbon monoxide (CO) has mainly gained infamy as a poisonous and silent killer. Recent evidence, however, demonstrates that CO may act as a critical signal involved in essential cellular physiological processes in both plants and animals [1]; for example, during inflammatory reactions, where it influences underlying processes, such as cell proliferation and the production of cytokines and growth factors [2,3,4]. Furthermore, CO mediates several plant physiological responses, such as seed germination, root development, induction of stomatal closure, increased tolerance to salt and heavy metals, as well as plant defense [5,6,7,8,9,10]. In addition, CO interacts with other signaling molecules, such as nitric oxide and reactive oxygen species (ROS), to regulate plant growth and development [10]. In both animals and plants, heme oxygenase (HO; EC1.14.99.3) is responsible for CO generation by catalyzing oxidative conversion of heme to CO, free iron, and biliverdin (BV), in the presence of molecular oxygen and electrons supplied by NADPH (reduced form of nicotinamide adenine dinucleotide phosphate) [1]. BV is then converted to the potent antioxidant bilirubin (BR) by biliverdin reductase [2]. In animals, so far, three isoforms of HO, namely HO1, HO2, and HO3, have been identified. Of these, HO1 is the main induced isozyme that generates CO in order to protect the tissue against a wide range of injuries [11]. In Arabidopsis, four HO genes have been characterized: HY1, HO2, HO3, and HO4 [12]. Similar to animal HO1, Arabidopsis HY1 is an inducible enzyme and participates in multiple stress-related and developmental processes; including UV exposure, salinity, drought, heavy metal exposure, oxidative insult, iron deficiency, and root organogenesis [10]. A function for HO1 has been identified in several plant species, such as medicago and barley, but its role and that of its product CO in tobacco remain less clear.

Tobacco (*Nicotiana tabacum*) can produce an array of alkaloids that defend against herbivore and insect attacks [13,14,15]. Nicotine is the main alkaloid produced by cultivated tobacco, constituting approximately 0.6–3% of tobacco leaf dry weight. Nicotine is synthesized in the root from the precursors ornithine and arginine via the intermediate compound putrescine [16]. In all higher plants, putrescine is either metabolized to higher polyamines, such as spermidine and spermine, or conjugated to either fatty acids or cinnamic acid derivatives; however, in plants that produce nicotine or tropane alkaloids, it may also be converted into *N*-methylputrescine. This step is catalyzed by putrescine *N*-methyltransferase (PMT; EC 2.1.1.53), which thus participates in the first committed step of alkaloid biosynthesis. *N*-methylputrescine may be oxidized and cyclized into l-methyl-A′-pyrrolinium cation by a diamine oxidase (EC 1.4.3.6), which is then converted into nicotinic acid or its derivatives by Quinolinic acid phosphoribosyltransferase (QAPRT; EC 2.4.2.19; an important enzyme in the pyridine nucleotide cycle). After biosynthesis in the tobacco root, nicotine is translocated to the leaf via the xylem and is stored in the leaf vacuole by a tonoplast-localized transporter [16,17]. The accumulation of nicotine in tobacco is affected by environmental factors and plant hormone levels. It has been reported that the phytohorome jasmonic acid (JA) significantly induces nicotine production in tobacco [18]. Biosynthesis of JA itself occurs in peroxisomes and is initiated from alpha-linolenic acid, which is converted into JA through a series of enzymatic reactions catalyzed by lipoxygenase (LOX), allene oxide synthase (AOS), allene oxide cyclase (AOC), and 12-oxophytodienoate reductase (OPR). Upon the activation of JA signaling, JA binds to the F-box protein COI1 to form an E3-ubiquitin ligase complex, stimulating degradation of the JA signal repressor JASMONATE ZIM-DOMAIN (JAZ) by the 26 S proteasome system and ultimately releasing the activity of MYC2 transcription factor(s) to activate JA-mediated defense responses. One such response is NtMYC2 upregulating putrescine *N*-methyltransferase (*NtPMT1*), the enzyme catalyzing the first committed step in nicotine biosynthesis. It has been reported that high temperature (HT) induces degradation of JASMONATE ZIM-DOMAIN proteins (JAZs) and increases NtMYC2 transcriptional activity, leading to increased nicotine biosynthesis in tobacco [18,19,20]. However, it remains less well understood how environmental stress affects nicotine biosynthesis in tobacco. 

In this study, we demonstrate that HT stress increases nicotine accumulation in tobacco roots, accompanied by a high level of JA biosynthesis. Furthermore, we found that HT stress increases HO enzyme activity, leading to increased CO production. Supplying CO-releasing molecules (CORM), such as tricarbonyl-dichlororuthenium (II) dimer (CORM-2), further increased JA and nicotine biosynthesis under HT conditions. By contrast, suppressing CO biosynthesis by treatment with the HO enzyme inhibitor ZnPPIX lowered both JA and nicotine levels. We found that HT promotes direct binding of NtMYC2a to the *NtPMT1* promoter, upregulating its expression and increasing nicotine biosynthesis. Based on these data, we propose that CO acts as a novel signal involved in HT-induced nicotine biosynthesis, possibly through a JA signal. Our study may inspire new research into how CO can be used to modify nicotine levels in tobacco plants.

## 2. Results

### 2.1. High Temperature Induces Nicotine Biosynthesis

**Figure 1:**
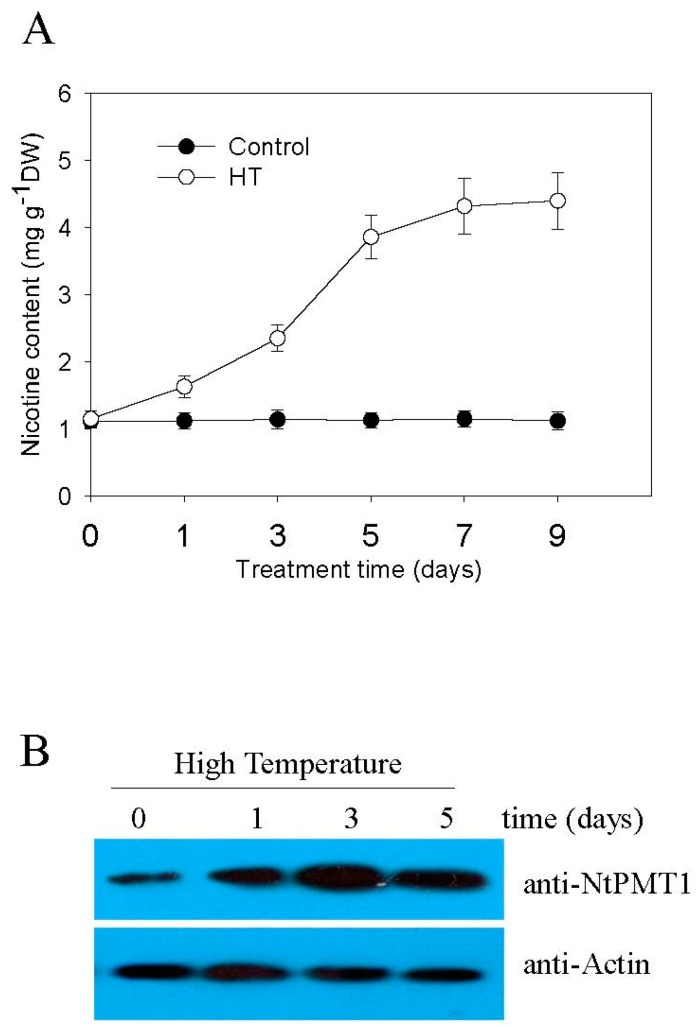
High temperature (HT) induces nicotine biosynthesis in tobacco roots. Two-week-old seedlings were treated with HT at 38 °C for the indicated time in days, seedlings grown at 24 °C were used as a control. At the indicated time, roots were collected and both nicotine content (**A**) and NtPMT1 protein content (**B**) in the root were analyzed. Data in (**A**) are the mean *±* SD *from* triplicate experiments (*n* = 6).

Nicotine is the main alkaloid produced in tobacco to defend against biotic stress, such as herbivore and insect attacks [13]. We therefore wondered whether abiotic environmental stress, such as high temperature, might similarly affect nicotine biosynthesis. First, we subjected plants to increasing high temperatures and measured the chlorophyll fluorescence to compare the effect on the leaf Fv/Fm ratio, Fv/Fm is the maxium quantum efficiency of cholorplast PSII system, Fv means the total amount of variable flurosence (dark adapted leaves), and Fm means the maximum fluorescence yield (dark adapted leaves, PSII fully closed). Fv/Fm is regards as a normalization index that reflects the efficiency of leaf photosynthesis. We found that the leaf Fv/Fm ratio under normal conditions at 24 °C was approximately 0.82, while increasing temperatures gradually reduced the leaf Fv/Fm ratio (Figure S1). At environmental temperatures over 40 °C, the leaf Fv/Fm ratio decreased to very low values (~0.16), therefore we opted to select a stress temperature of 38 °C, at which point, the Fv/Fm ratio measured ~0.50 (Figure S1). We then proceeded to subject two-week-old tobacco seedlings to HT at 38 °C, and subsequently measured their root nicotine content. As shown in Figure 1A, HT treatment induces gradual nicotine accumulation, which reaches a plateau level after seven days of treatment. Consistent with this, HT treatment also stimulates gradual accumulation of NtPMT1, a key enzyme involved in synthesizing nicotine, in root tissue (Figure 1B), confirming that HT induces nicotine biosynthesis in tobacco seedling roots.

### 2.2. High Temperature Induces CO and JA Biosynthesis

**Figure 2:**
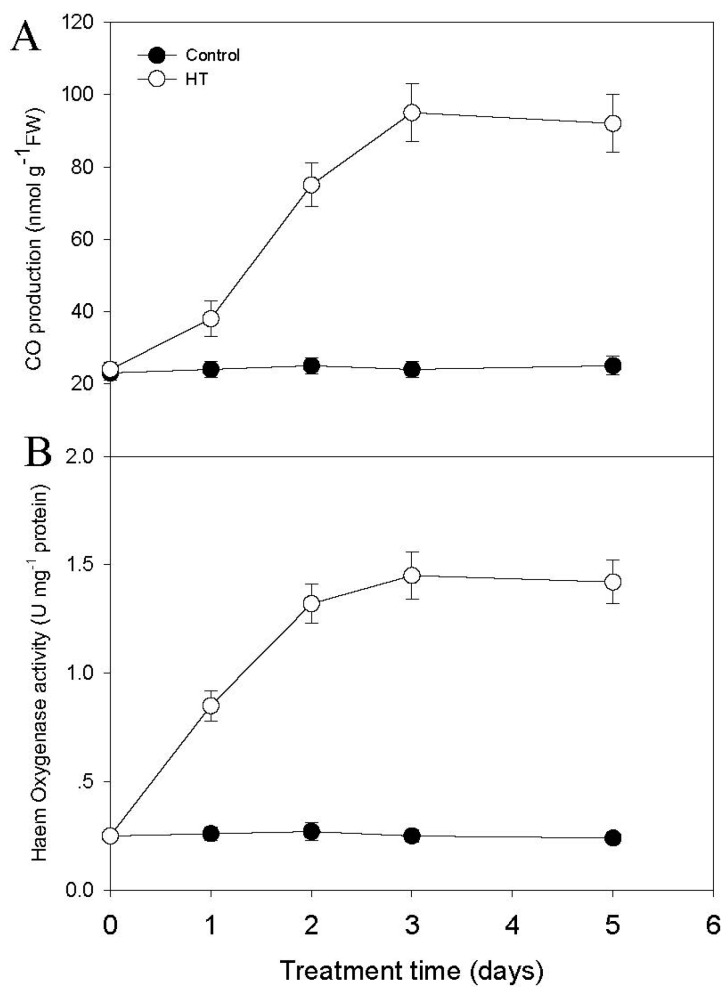
HT induces CO accumulation and HY1 activity in tobacco seedlings. Two-week-old seedlings were treated with HT at 38 °C, the seedling under 24 °C were used at the control. At the indicated time, both CO level (**A**) and HY1 activity (**B**) in the root were analyzed. Data are the mean *±* SD from triplicate experiments (*n* = 6).

The small molecule CO is involved in many plant physiological responses to environmental stress [10], and heme oxygenase (HY) is the main enzyme responsible for CO biosynthesis in the plant [10,11]. We therefore aimed to study whether HT might induce activity of the CO biosynthesis enzyme HY, and thereby increase CO generation in tobacco roots. As shown in Figure 2A, HT induced CO production in tobacco roots, reaching its plateau level after three days of treatment. HO enzyme activity was also induced by HT treatment, showing a similar pattern as for CO generation (Figure 2B). These data demonstrate that HT induces both CO production and an increase in HO enzyme activity.

**Figure 3:**
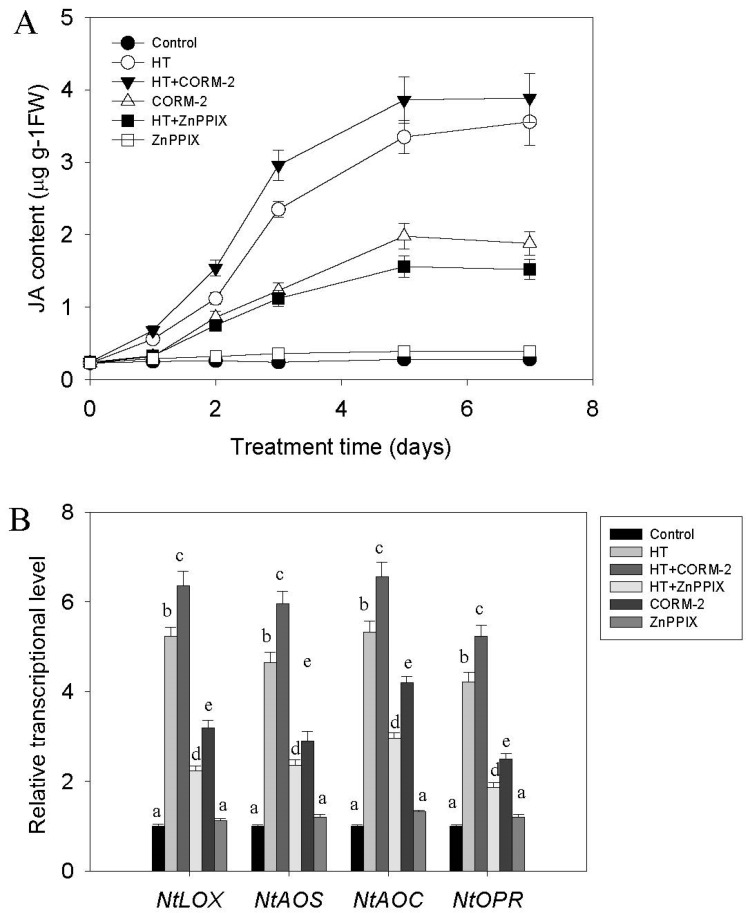
HT and CO induce JA accumulation and expression of JA biosynthesis related genes in tobacco seedlings. Two-week-old seedlings were treated with HT and CO or ZnPPIX at 38 °C for the indicated time, after which JA content was measured. For CO or ZnPPIX treatment, the CO donor CORM-2 (10 nM), ZnPPIX (100 µM) were added respectively to the culture solution following HT treatment, the sample treated with CORM2 or ZnPPIX under normal temperature conditions was regarded as the negative control. JA content was measured at the indicated time (**A**), and the transcriptional levels of JA biosynthesis related genes, including *NtLOX, NtAOS, NtAOC, NtOPR* were measured after 12 h of HT treatment by QRT-PCR analysis (**B**). Data plotted are the mean *±* SD from triplicate experiments (*n* = 6). Bars with different letters between the treatment sample in the same group represent statistically significant difference at *p* < 0.05 (Tukey’s test).

Previous studies have shown that JA signaling may directly control nicotine biosynthesis [18], so we investigated whether HT treatment might also increase tobacco root JA content. As shown in Figure 3A, JA content could gradually be induced by HT stress, and reached its peak level after five days of HT treatment. Correspondingly, the level of transcription of the JA biosynthesis related genes *NtLOX*, *NTAOC*, *NtAOS*, and *NtOPR* was also increased (Figure 3B). As HT induces accumulation of both CO and JA, we wondered whether the CO signal might be responsible for regulating JA biosynthesis in response to HT stress. To test this, we treated tobacco seedlings with the CO donor CORM-2 or the HO enzyme inhibitor zinc protoporphyrin IX (ZnppIX), while subjecting the plants to HT stress. We found that additional CORM-2 treatment markedly increased, while ZnPPIX treatment suppressed HT induction of both JA biosynthesis and transcriptional levels of genes involved in its biosynthesis (Figure 3A,B).

### 2.3. CO Signaling Potentiates HT-Induced Nicotine

**Figure 4:**
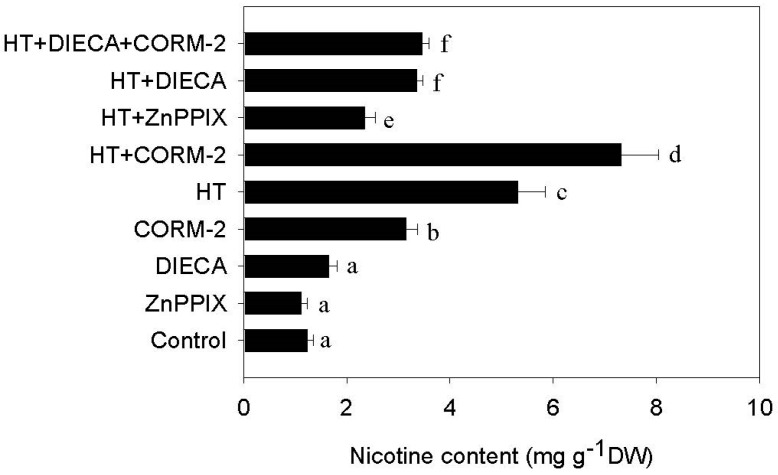
CO signaling increases nicotine biosynthesis in tobacco roots under HT. Two-week-old seedlings were treated with HT at 38 °C for five days, after which nicotine content was measured. For CO, ZnPPIX or DIECA treatment, the CO donor CORM-2 (10 nM), ZnPPIX (100 µM) or DIECA (1 mM) were added, respectively, to the culture solution following with HT treatment. These chemicals being added under normal temperature conditions were used as the negative control in each case. Data plotted are the mean ± SD from triplicate experiments (*n* = 6). Bars with different letters represent groups that have a statistically significant difference from the other groups at *p* < 0.05 (Tukey’s test).

Because HT induces CO and nicotine biosynthesis (Figure 2A), as well as JA accumulation (Figure 3A), it is possible that the CO signal heightens the level of nicotine biosynthesis via an increase in JA signaling. To investigate this, we pretreated tobacco seedlings with the CO biosynthesis inhibitor ZnPPIX, and followed up with HT stress treatment. We found that ZnPPIX treatment significantly suppressed the HT-induced increase in nicotine content (Figure 4). By contrast, adding CORM-2 further increased HT-induced nicotine biosynthesis levels (Figure 4). However, treating tobacco seedlings with the JA biosynthesis inhibitor sodium diethyldithiocarbamate trihydrate (DIECA) could suppress HT-induced nicotine biosynthesis, while adding CORM-2 did not counteract the inhibitory effect of DIECA on HT-induced nicotine biosynthesis (Figure 4). These data suggest that increased levels of the small molecule CO transduce part of the HT induced increase in nicotine biosynthesis, which is likely to be mediated via an increase in JA biosynthesis and signaling.

### 2.4. CO Signaling Increases the Transcriptional Activity of MYC2a under HT Stress

**Figure 5:**
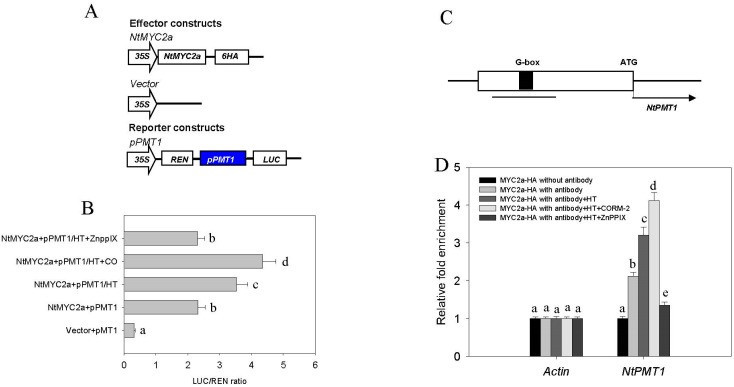
CO signaling increases *NtMYC2a*-mediated *NtPMT1* transcription under HT. (**A**,**B**) Transient expression assays performed in Arabidopsis mesophyll protoplasts to test the effect of CO signaling on *NtPMT1* transcription. (**A**) Schematic diagrams of the constructs used in the transient expression assays in; The blue box means the promoter region of *PMT1* gene (**B**) the bar graph shows the LUC/REN ratio that results from expressing the specific constructs in each experiment. Vector+pPMT1: co-transformation with empty vector and pPMT1 reporter vector; NtMYC2a+pPMT1: co-transformation with NtMYC2a vector and pPMT1 reporter vector; NtMYC2a+pPMT1/HT: co-transformation with NtMYC2a vector and pPMT1 reporter vector under 38 °C; NtMYC2a+pPMT1/HT+CO: co-transformation with NtMYC2a vector and pPMT1 reporter vector under 38 °C with 10 nM CORM-2; NtMYC2a+pPMT1/HT+ZnPPIX: co-transformation with NtMYC2a vector and pPMT1 reporter vector under 38 °C with 100 µM ZnPPIX. Data plotted are the mean ± SD from triplicate experiments (*n* = 3). Bars with different letters represent groups that have a statistically significant difference from the other groups at *p* < 0.05 (Tukey’s test); (**C**,**D**) ChIP analysis of MYC2a binding to the G-box region in the *NtPMT1* promoter in MYC2a-GFP transgenic seedlings; (**C**) schematic representation of the *NtPMT1* promoter, with the black box representing the G-box where NtMYC2 binds and the black line below the NtPMT1 promoter indicating the region amplified for the ChIP qRT-PCR assay; (**D**) two-week-old seedlings were treated with CORM-1 or ZnPPIX under HT stress, ChIP was done using an anti-HA antibody, after which qRT-PCR was performed on the *NtPMT1* G-Box. A fragment of the *Actin* promoter was used as negative control (**D**). Data plotted are the mean ± SD from triplicate experiments (*n* = 3). Bars with different letters represent groups that have statistically significant difference from the other groups at *p* < 0.05 (Tukey’s test).

In tobacco, nicotine biosynthesis is directly controlled by expression of the NtNPT1 enzyme. The NtMYC2 bHLH transcription factor can recognize and bind to the core region of the NtNPT1 promoter to control its expression. NtMYC2 is normally inhibited by the NtJAZ protein, but increased JA levels can promote NtJAZ degradation to activate NtMYC2, thereby promoting nicotine biosynthesis [18,20,21]. Previous studies have demonstrated that HT, like JA, promotes the degradation of NtJAZ1 to release NtMYC2a activity and increase the NtPMT1 transcriptional level [22]. To test whether the CO signal is also required for the HT-induced transcriptional increase of NtPMT1 through NtMYC2a, we used a dual-luciferase reporter approach to analyze the effect of CO or HT treatment on MYC2a-activated transcription of *NtNPT1* in tobacco root cell protoplasts. In this assay, the reporter consists of cauliflower mosaic virus *35S* promoter-driven Renilla luciferase (*35S::REN*, used as a control for transfection efficiency) and *NPT1* promoter-driven firefly luciferase (*pNPT1::LUC*). The effector constructs for *NtMyc2a* were expressed under the control of the 35S promoter. As shown in Figure 5B, HT treatment increases the LUC/REN ratio in protoplasts transiently co-expressing *35S:NtMYC2a* and *pNtPMT1*. This effect could be further enhanced by additional CO donor CORM-2 treatment, or suppressed by HO enzyme inhibitor ZnPPIX treatment. These data suggest that CO enhances the HT-induced level of *NtPMT1* transcription in vitro. Previous studies have shown that a so-called G-box (GCACGTTG) in the promoter region of NtPMT1 is characterized as the MYC2a binding site for JA induced nicotine biosynthesis (Figure 5C) [21,23]. We performed a ChIP analysis to test how strongly *NtMYC2a* would bind to the *NtPMT1* promoter in response to HT and CO. As shown in Figure 5D, HT treatment markedly increased binding of NtMYC2a to the *NtPMT1* promoter, and supplying additional CO via the CORM-2 donor further enhanced this binding, while co-treatment with the CO biosynthesis inhibitor ZnPPIX reduced NtMYC2a binding. These data further confirm the synergistic effect of CO and HT stress on transcription of *NtPMT1*, and suggest that CO likely acts to enhance the binding of NtMYC2a to the *NtPMT1* promoter, leading to increased nicotine biosynthesis under HT stress.

## 3. Discussion

Like in animals, CO has also been found to be an essential signal for regulating a series of physiological processes in plants [5,6,7,8,9,10]. Different environmental stresses, including high salt, heavy metals, iron deficiency, and temperature stress, can induce CO accumulation [8,9]. For example, in *Baccaurea ramiflora* seeds, cold stress may induce the transient accumulation of CO, while exogenous application of CO could enhance seed tolerance to cold stress [24]. In *Medicago sativa*, increasing temperature leads to enhanced HY1 activity, promoting CO production [25]. In renal epithelial cells heme oxygenase activity can be modulated by changing the ambient temperature [26]. Consistent with this, here we show that HT can similarly induce CO generation in tobacco seedlings via increased activity of the NtHY1 enzyme (Figure 2). Previous studies have shown that HT stress also induces nicotine biosynthesis in tobacco seedling through another small molecular signal hydrogen sulfide [27]. Like hydrogen sulfide, CO also acts as a novel signal to mediate nicotine biosynthesis under HT stress, since directly applying CO via the CO artificial donor CORM-2 could induce nicotine biosynthesis. By contrast, suppressing NtHY1 enzyme activity by ZnPPIX treatment compromised HT-induced nicotine biosynthesis (Figure 4).

Accumulating evidence supports that environmental temperature change also affects JA biosynthesis and modulates the plant defense response. For example, cold stress can increase JA content in Arabidopsis and rice, while the application of exogenous JA enhances their tolerance to freezing stress [28,29]. Similarly, the involvement of JA signaling in regulating thermotolerance has been reported recently; heat stress leads to the accumulation of several jasmonates, including OPDA, MeJA, JA, and JA-Ile. In addition, exogenous JA treatment maintains cell viability under HT conditions, while Arabidopsis mutants deficient in JA signaling, such as *coi1-1* and *opr3*, show increased sensitivity to heat stress [30]. In tobacco, HT promotes JA biosynthesis and the subsequent degradation of NtJAZ1, leading to the release of NtMYC2a, which is then able to upregulate NtPMT1 transcription and drive nicotine biosynthesis [31]. In agreement with this, we also found that HT stress increases tobacco root JA content. We expand upon this finding by showing that HT-induced JA biosynthesis can be suppressed by reducing CO biosynthesis through the ZnPPIX inhibitor. Furthermore, treating tobacco with the CO donor CORM-2 directly induces the accumulation of JA through upregulating the transcriptional levels of JA biosynthesis related genes; including *NtLOX, NtAOS, NtAOC* and *NtOPR* (Figure 3B). These results suggest a novel role for CO in mediating HT-induced JA biosynthesis. CO is not the only small gas molecule that activates JA signaling, since nitric oxide (NO) was found to similarly enhance the plant defense response [30,32,33]. Since previous studies have shown that the biological function of CO on iron uptake was associated with NO signal [22,34], it is possible that CO interacts with the NO signal to activate JA biosynthesis under HT stress. JA plays a critical role in inducing the biosynthesis of secondary metabolites in several plants [35], such as nicotine biosynthesis in tobacco [18,20,21,23]. Here, we found that HT-induced nicotine biosynthesis depends on a JA signal, because suppressing JA biosynthesis by DIECA reduced HT-induced nicotine biosynthesis. Furthermore, under HT stress, this inhibitory effect could not be efficiently recovered by supplying additional CO (Figure 4). These data indicate that CO likely acts upstream of the JA signal controlling nicotine biosynthesis, and suggest that its role is to directly induce JA biosynthesis.

Under normal conditions, NtMYC2a is sequestered by NtJAZ1, leading to a lower level of NtPMT1 transcription and nicotine biosynthesis. Once tobacco is subjected to HT, increased levels of JA cause the degradation of NtJAZ1, releasing NtMYC2a, which then upregulates *NtPMT1* transcription, increasing nicotine biosynthesis [31]. In agreement with this, our data show that suppressing the JA signal by DIECA compromises HT-induced nicotine biosynthesis. In addition, using transient protoplast transformation, we found that ZnPPIX treatment impaired the HT-induced transcriptional upregulation of *NtPMT1*. Furthermore, treatment with the CO donor CORM-2 following HT-stress increased transcriptional output of the *NtPMT1* gene, suggesting the CO and JA signal to coordinately mediate HT-induced nicotine biosynthesis. Finally, our ChIP analysis showed that HT treatment accelerated binding of NtMYC2a to the promoter region of *NtPMT1* under HT stress, an effect that could be strengthened by the HT-induced CO signal. These data further corroborate our finding that HT induces nicotine biosynthesis through CO.

**Figure 6:**
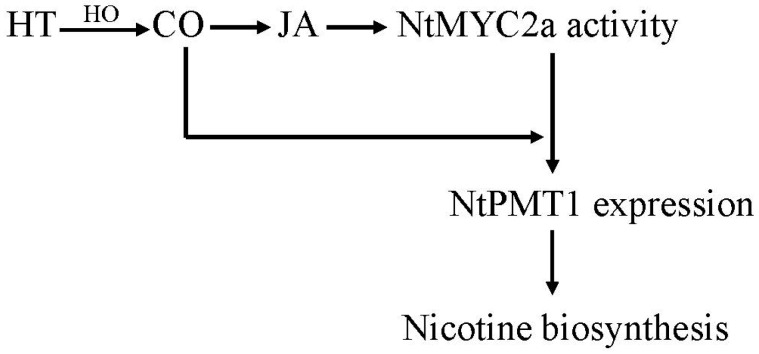
Our proposed model to illustrate how CO regulates HT induced nicotine biosynthesis in tobacco roots. After plants are subjected to HT, HO-dependent CO biosynthesis is increased, which leads to triggering of a JA signal that activates NTMYC2a on the one hand, and CO-enhanced binding of NtMYC2a to the NtPMT1 promoter on the other hand, finally resulting in increased nicotine biosynthesis under HT stress.

Taken together, in this study we have reported a new function for CO in potentiating HT-induced nicotine biosynthesis in tobacco. Based on our findings, we propose a model to highlight the role of CO in nicotine biosynthesis (Figure 6). In this model, HT stress leads to increased levels of NtHY1 enzyme, subsequently leading to more CO production in the plant. Higher CO levels trigger the accumulation of JA and subsequent degradation of NtJAZ1. The degradation of NtJAZ1 releases NtMYC2a from its inhibitor, allowing for it to bind to the NtPMT1 promoter, activating its expressing and increasing nicotine biosynthesis. It is not yet clear that the physiological relevance increased nicotine production in response to heat stress. However, one possible hypothesis is that an increased ambient temperature (which, incidentally, is also predicted to result from the current global warming trend) heightens the frequency of insect attacks [36]. JA-induced nicotine is a critical compound for tobacco to defend against insect attacks [31], thus our finding highlights the important role of HT-induced CO signaling and nicotine biosynthesis in enhancing the ecological adaptation of tobacco to climate warming. Thus, our findings provide new insights that can be used to improve nicotine biosynthesis through modifying CO signaling in tobacco. 

## 4. Materials and Methods

### 4.1. Plant Growth and High Temperature Treatment

Tobacco (*Nicotiana. tabacum cv*. Wisconsin 38) seeds were sterilized and germinated, then grown to seedlings under constant white light on half-strength Gamborg B5 medium supplemented with 2% (*w*/*v*) gellan gum and 0.3% sucrose at 24 °C. One-week-old plants were transferred to hydroponic holders filled with nutrient solution, consisting of 300 µM Ca(NO_3_)_2_, 50 µM MgSO_4_, 30 µM NaH_2_PO_4_, 50 µM K_2_SO_4_, 3 µM H_3_BO_3_, 0.4 µM ZnSO_4_, 0.2 µM CuSO_4_, 0.5 µM MnCl_2_, 1 µM (NH_4_)_6_Mo_7_O_4_, and 20 µM Fe-EDTA at pH 6.5 (KOH), for another one week of culture. To perform high temperature treatment, two-week-old seedlings were shifted to a constant 38 °C (16 h light/8 h dark, 1200, 450 μmol·m^−2^·s^−1^ light intensity), with control seedlings left at 24 °C. For treatment with JA and other chemicals, in all the cases, these were dissolved in 1% DMSO (dimethyl sulfoxide) as the stock solution, then added into the nutrient solution at the indicated working solution (controls were treated with only 1% DMSO). For both experiments, following a specific time of treatment, tobacco roots were collected directly and processed for further analysis. 

### 4.2. Nicotine Content Analysis

Nicotine content was measured as previously described [27]. In brief, after each treatment, 0.5 g of tobacco roots was collected and washed clean, homogenized in liquid nitrogen, after which the powder was dissolved in 0.1 M H_2_SO_4_ and sonicated for 60 min. After centrifugation at 2000× *g* for 15 min, the supernatant was collected and 0.4 mL 25% NH_4_OH was added to neutralize the acidic solution. The mixture was then loaded onto an Extrelut-1 column (Merck, Darmstadt, Hesse, Germany) and eluted using 6 mL of chloroform. The eluent was dried under 37 °C, then dissolved in ethanol, and subsequently analyzed by both gas chromatography (Agilent 6890) and quadrupole mass spectrometry with electron impact ionization (Agilent 5973, Network, Wilmington, DE, USA). Column temperature was held at 100 °C for 10 min, then increased to 260 °C during a 35-min period at a gradient of 8 °C/min. Artificial nicotine, obtained from the Zhengzhou Tobacco Research Institute, was used as a standard.

### 4.3. Jasmonic Acid Content Analysis

Jasmonic acid content was determined, as previously described [37]. In brief, 200 mg of tobacco root after various treatments was collected and quickly ground under liquid nitrogen. 1.5 mL of methanol containing 60 ng of [^2^H_6_](±)-JASMONIC ACID (D-JA) [3-oxo-2-([3-^2^H,4-^2^H_2_,5-^2^H_3_](Z)-2-pentenyl)cyclopentane-1-acetic acid] (Olchemlm LTD, Olomouc, Czech Republic) was added into the mixture as an internal standard. JA content was then measured by high performance liquid chromatography-tandem mass spectrometry (HPLC-MS/MS, ThermoFisher Scientifc, Waltham, MA, USA), as previously described [37].

### 4.4. RNA Extraction and Quantitative Real-Time RT-PCR (qRT-PCR)

Total RNA was extracted from 3-week-old tobacco seedlings using the TRIzol reagent (Invitrogen, ThermoFisher Scientifc, Waltham, MA, USA) and qRT-PCR was performed as previously described [38]. Briefly, first-strand cDNA was synthesized by M-MuLV reverse transcriptase (Fermentas, ThermoFisher Scientifc, Waltham, MA, USA) using DNase-treated RNA and oligo (dT)_18_ primer. For real-time PCR, cDNA samples were diluted to 2–10 ng/mL, and PCR was performed using the SYBR Green I Master Mix with a Roche LightCycler 480 real-time PCR machine, according to the manufacturer’s instructions. At least three biological replicates for each sample were analyzed in each qRT-PCR experiment and at least two technical replicates were analyzed for each biological replicate. *ACTIN2* was used as an internal gene expression control. Gene-specific primers used to detect each transcript are listed in S1.

### 4.5. Protein Isolation and Immunoblot Analysis

Proteins were extracted using protein extraction buffer (50 mM Tris-Cl, pH 7.5, 150 mM NaCl, 1 mM PMSF, 1× Complete Protease Inhibitor Cocktail (Roche, Pleasanton, CA, USA), 5% glycerol, 1 mM EDTA, 1 mM DTT), and protein concentration was measured by a commercial Bradford assay kit, following the manufacturer’s instructions (Bio-Rad, Hercules, CA, USA) After 12% SDS-PAGE electrophoresis, fractionated proteins were transferred to a PVDF membrane and immunoblot assays were performed with the ECL method [22], using anti-PMT1 and anti-Actin (Abmart, Shanghai, China) antibodies at dilutions of 1:3000 and 1:2000, respectively. The anti-PMT1 antibody was prepared by immunizing one rabbit, as previously described [22].

### 4.6. CO and Heme Oxygenase (HO) Activity Analysis

Carbon monoxide (CO) in tobacco roots was quantified using a previously described method [22]. Briefly, 0.5 g of treated roots was ground into fine powder using liquid nitrogen and transferred into a 4 mL bottle. Samples were stored under vacuum in an ultralow chiller at −70 °C prior to further processing. To avoid foaming, about 1 mL distilled water containing 20 μL 1-octanol was sequentially added to each bottle. After shaking vigorously for approximately 30 s, 1 mL of sulfuric acid was added into each bottle through the rubber cap using a syringe with a needle. The bottles were then kept into a 70 °C water bath for 3 h, and subsequently cooled down to room temperature for analysis. CO concentration was quantified by GC-MS analysis using 1 mL of air from the headspace. HY1 enzyme activity was determined, as previously described [24].

### 4.7. Transient Protoplast Transformation

A 3000-bp *NtPMT1* 5′ upstream promoter fragment was amplified from genomic DNA and inserted into pGreenII 0800-LUC to generate the *P_PMT1_-LUC* reporter construct. The coding sequence of *NtMYC2a* was amplified and inserted into the pGreenII 62-SK vector under control of the 35S promoter. These constructs were subsequently used to perform transient transcription activity assays using the LUC reporter (all primers used are listed in Table S1). Tobacco root cell protoplasts were prepared and transiently transfected, as previously described [39]. Relative REN activity was used as an internal control. LUC/REN ratios were calculated.

### 4.8. Chip Assay

The CHIP assay was manipulated as previous method using 100 mg of tobacco root with or without HT stress [31]. Immunoprecipitation was performed with rabbit polyclonal antibody against HA (1:1000, clone 3F10, Roche). NtPMT1 region were detected using the PCR primers listed in Table S1. Chip products were analyzed by quantitative PCR. Data from the Chip experiments are expressed as the mean ± SD of three biological replicates.

## Supplementary Materials

Supplementary materials can be found at www.mdpi.com/1422-0067/19/1/188/s1.
