# Supplementary material for: Carbon Monoxide Potentiates High Temperature-Induced Nicotine Biosynthesis in Tobacco
Source: Int J Mol Sci. 2018 Jan 8;19(1):188. doi: 10.3390/ijms19010188 (PMC5796137; doi:10.3390/ijms19010188)
Supplement: Supplementary file 1 [file ijms-19-00188-s001.zip › Supplemental Figure 1_comms Remco.pdf]

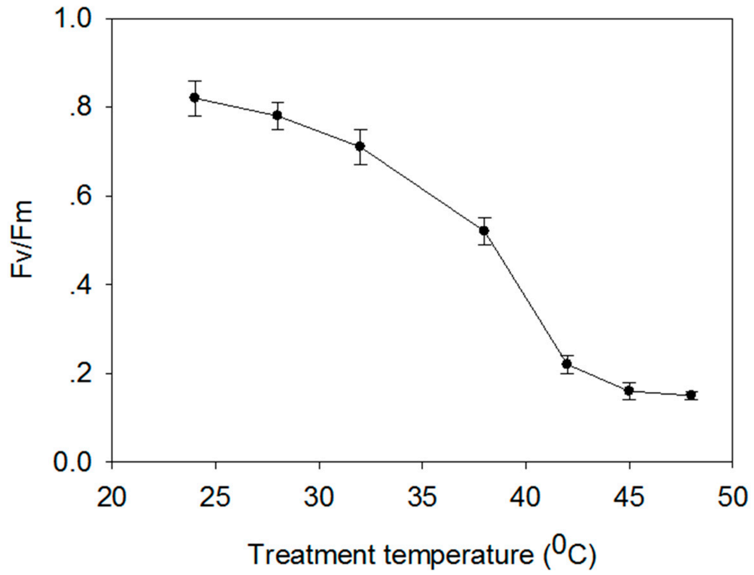

Figure S1. High temperature decreases leaf photosynthesis efficiency in tobacco seedlings. Two-week-old tobacco seedling were placed at the indicated temperature, after three days of treatment the leaf photosynthesis Fv/Fm ratio was determined. Data plotted are the mean ± SD from triplicate experiments (n = 3).
